# Concomitant or late aortic valve intervention and its efficacy for aortic insufficiency associated with continuous-flow left ventricular assist device implantation

**DOI:** 10.3389/fcvm.2022.1029984

**Published:** 2022-11-15

**Authors:** Masahiko Ando, Minoru Ono

**Affiliations:** Department of Cardiac Surgery, The University of Tokyo Hospital, Tokyo, Japan

**Keywords:** left ventricular assist device (LVAD), aortic insufficiency (AI), aortic valve replacement (AVR), aortic valve repair (AV repair), heart transplant (HTx)

## Abstract

Moderate to severe aortic insufficiency (AI) in patients who underwent continuous-flow left ventricular assist device (CF-LVAD) implantation is a significant complication. According to the INTERMACS registry analysis, at least mild AI occurs in 55% of patients at 6 months after CF-LVAD implantation and moderate to severe AI is significantly associated with higher rates of re-hospitalization and mortality. The clinical implications of these data may underscore consideration of prophylactic aortic valve replacement, or repair, at the time of CF-LVAD implantation, particularly with expected longer duration of support and in patients with preexisting AI that is more than mild. More crucially, even if a native aortic valve is seemingly competent at the time of VAD implantation, we frequently find *de novo* AI as time goes by, potentially due to commissural fusion in the setting of inconsistent aortic valve opening or persistent valve closure caused by CF-LVAD support, that alters morphological and functional properties of innately competent aortic valves. Therefore, close monitoring of AI is mandatory, as the prognostic nature of its longitudinal progression is still unclear. Clearly, significant AI during VAD support warrants surgical intervention at the appropriate timing, especially in patients of destination therapy. Nonetheless, such an uncertainty in the progression of AI translates to a lack of consensus regarding the management of this untoward complication. In practice, proposed surgical options are aortic valve replacement, repair, closure, and more recently transcatheter aortic valve implantation or closure. Transcatheter approach is of course less invasive, however, its efficacy in terms of long-term outcome is limited. In this review, we summarize the recent evidence related to the pathophysiology and surgical treatment of AI associated with CF-LVAD implantation.

## Introduction

Moderate to severe aortic insufficiency (AI) in patients who underwent continuous-flow left ventricular assist device (CF-LVAD) implantation is a significant complication affecting long-term outcomes (1–7). According to the INTERMACS registry analysis, at least mild AI occurs in 55% of patients at 6 months after CF-LVAD implantation and moderate to severe AI is significantly associated with higher rates of re-hospitalization and mortality (1). The clinical implications of these data may underscore consideration of prophylactic aortic valve replacement (AVR), or repair, at the time of CF-LVAD implantation, particularly with expected longer duration of support and in patients with preexisting AI that is more than mild (8). More crucially, even if a native aortic valve (AV) is seemingly competent at the time of VAD implantation, we frequently find *de novo* AI as time goes by, potentially due to commissural fusion in the setting of inconsistent aortic valve opening or persistent valve closure caused by CF-LVAD support, that alters morphological and functional properties of innately competent aortic valves (9–13). Therefore, close monitoring of AI is mandatory, as the prognostic nature of its longitudinal progression is still unclear. Clearly, significant AI during CF-LVAD support warrants surgical or percutaneous intervention at the appropriate timing (14–18), especially in patients of destination therapy. Nonetheless, such an uncertainty in the progression of AI translates to a lack of consensus regarding the management of this untoward complication. Additionally, before facing to the evaluation of AI during CF-LVAD support, even today, we have not yet established a reliable, or reproducible, method of quantifying the grade of AI in those patients. In the patients under CF-LVAD support, color doppler method might not be enough to measure the amount of actual AI regurgitant flow, as in most circumstances, significant CF-LVAD-associated AI is a continuous one, not a diastolic one, due to continuous suction by the devices. Such another uncertainty, or variability, in the evaluation of AI might have partly contributed to a current lack of consensus in this topic.

In practice, proposed surgical options are AVR (19, 20), AV repair (21–28), AV closure (29), and more recently transcatheter aortic valve implantation (TAVI) (30–33) or transcatheter device closure (34, 35). Briefly, AVR with bioprosthetic valve could be a gold standard treatment for AI in CF-LVAD patients, especially when the native AV contains structural problems. However, it necessitates longer ischemic time, posing a concern of further deterioration of biventricular function in these VAD patients particularly with reduced right ventricular function at baseline. AV repair, or what we call central AV closure (CAVC) or Park's stitch (21, 22), is more simple and technically possible with shorter ischemic time under limited AV exposure. The drawback of CAVC is a durability specifically when the patients' expected support duration is long, such as destination therapy. AV closure could be a last option to consider, as the clinical outcome is not satisfactory (29). Finally, transcatheter approach is of course less invasive, however, its efficacy in terms of long-term outcome is limited. In this review, we summarize the recent evidence related to the pathophysiology and surgical treatment of AI late after CF-LVAD implantation.

## Pathophysiology of AI associated with CF-LVAD support

Although the true mechanisms of *de novo* AI under CF-LVAD support remain controversial, following three factors are likely to be associated with *de novo* AI: (1) continuous or intermittent AV closure due to the constant increase of aortic diastolic pressure with the decrease in LV end-diastolic pressure, (2) increased transvalvular gradient due to decompression of the LV, leading to stretching or partial prolapse of AV leaflets, and (3) pathologic changes or dilatations in the aortic sinus due to turbulent backflow with high blood velocity from a CF-LVAD outflow (11, 12). These factors could interact with one another, eventually yielding AV disorganization and/or commissural fusion, with a time-related manner. Historically, as diagnostic modalities were quite limited, the effect of CF-LVAD on aortic blood flow dynamics and kinetics as well as on AV physiology had not been fully elucidated. Today, computational fluid dynamic (CFD) studies have demonstrated that the blood stream from LVAD outflow could increase the shear stress on the aortic root and AV. Kasinpila et al. conducted a CFD study in 10 patients with *de novo* AI and 20 patients without AI after CF-LVAD implantation, and concluded that those who developed *de novo* AI had greater wall shear stress on the aortic root and their outflow grafts were placed closer to the aortic root than those patients without *de novo* AI (13). Similar CFD studies were reported by Yoshida et al. (9). They investigated the impact of non-physiological retrograde blood flow in the aortic root on *de novo* AI after CF-LVAD implantation by CFD analysis. Yoshida et al. demonstrated that those with *de novo* AI had a perpendicular outflow anastomosis at the ascending aorta, concluding the angle and position of LVAD outflow anastomosis might impact retrograde blood flow and *de novo* AI after CF-LVAD implantation (9). While higher wall shear stress on the aortic root could be associated with root or annular dilatation, eventually leading to AI progression, lower wall shear stress, as compared to physiological one, is known to be a cause of atherosclerosis (36). Based on the idea, Kainuma et al. proposed a different explanation on the AV degeneration during CF-LVAD support (10). They used an intraoperative epi-aortic echocardiography and calculated wall shear stress by vector flow mapping technology. This *in-vivo* study, not a computational simulation, demonstrated peak wall shear stress on the ascending aorta, aortic root, and ventricularis of AV was significantly reduced by CF-LVAD support, as compared to baseline (before LVAD). Kainuma et al. suggested such an altered mechanical stress on the AV could be associated with the structural, functional, and histological changes of the aorta and AV (10). Thus, we need more prospective studies to fully clarify the true mechanism of LVAD-induced AI.

## Incidence and clinical significance of late AI during CF-LVAD support

In contrast to the pathophysiology, the incidence of late AI during CF-LVAD support has been well-documented (1, 37). A recent analysis on the INTERMACS registry revealed late AI as a progressive disease that develops during CF-LVAD support with well-over 50% developing mild disease at 6 months of support and 15% developing moderate to severe within 2 years (1). Predictors of worsening AI included older age, female sex, smaller body mass index, mild pre-implantation AI, and destination therapy. Significant AI was associated with higher rates of rehospitalization (32.1 vs. 26.6%, *p* = 0.015) and lower rates of survival (77.2 vs. 71.4%, *p* = 0.005) (1). There are a few other recent single-institutional studies focused on detrimental effects of AI after CF-LVAD (2–6). Auvil et al. reported that they found moderate or greater AI in 8.5% of patients who underwent CF-LVAD implantation, at 6 months after the implant, and demonstrated that moderate AI was significantly associated with 2-year mortality after the implant [Odds ratio (OR) 4.32, 95% CI 1.21–15.4, *p* = 0.024] (4). Imamura et al. reported that worsening of AI was observed 53.7% of CF-LVAD patients at 3 months after the implantation, which was significantly associated with higher hazard of death or heart-failure readmission (HR 3.24, 95% CI 1.02–18.5, *p* = 0.038) (2). Kagawa et al. reported that 13.3% of CF-LVAD patients progressed to significant AI during median follow-up of 469 days, and mortality during the follow-up was significantly higher in the significant AI group (59.5 vs. 37.2%, *p* = 0.006) (5).

In contrast, there are several studies proposing that the influence of late AI on mortality during CF-LVAD support is not significant. Patil et al. reported that mild AI developed in 51.6% of CF-LVAD patients over a median duration of 126 days and moderate one developed in 14.0% over a median duration of 493 days (37). Like other studies, independent predictors of AI were duration of support and persistently closed aortic valve, although they did not find any association between AI progression and survival outcomes. Holley et al. showed that significant *de novo* AI occurred in 15.2% of patients after CF-LVAD implantation and such a *de novo* AI was not significantly associated with mortality (38). Compatible to the prior studies, they concluded that the independent predictors of late AI were older age, female gender, longer duration of LVAD support, and destination therapy.

As for the predictors of late AI, the effect of device type is a matter of much account and still controversial (18, 37, 39, 40). Historically, the development of intermittent low-speed (ILS) algorithm, or its analog, to avoid persistent closure of the AV, was expected to decrease the rate of late *de novo* AI (41–45). However, the favorable evidence of its efficacy on late AI is still limited. Patil et al. compared 58 HeartMate II (Axial pump, Abbott, MN, USA) cases with 35 HeartWare HVAD (Centrifugal pump, Medtronic, MN, USA) cases, and reported that the incidence of mild or greater AI was 43.1% in HeartMate II vs. 65.7% in HeartWare HVAD (*p* = 0.035, without baseline adjustment), during median follow-up of 527 days (37). Malic et al. compared 270 HeartMate II cases with 121 HeartMate 3 (Abbott, MN, USA), and reported that the cumulative incidence of mild or greater AI was 11.3% in HeartMate II vs. 8.4% in HeartMate 3 (*p* = 0.68, with baseline adjustment), at 1 year after VAD implantation (39). Finally, Jimenez Contreras et al. compared 562 HeartMate II cases with 300 HeartMate 3 cases, and reported that the incidence of moderate or severe AI was 17.0% in HeartMate II vs. 9.9% in HeartMate 3 at 6 months after VAD implantation. The multivariable Cox regression analysis demonstrated that the adjusted HRs of moderate or severe AI in HeartMate 3, as compared to HeartMate II, was 0.624 (*p* = 0.0537, 95% CI 0.386–1.008) (40). More recently, Uriel et al. presented based on MOMENTUM 3 pivotal trial (42), that the incidence of moderate or severe AI was 11.5% in HeartMate II vs. 5.6% in HeartMate 3 at 2 years after VAD implantation and the HRs of moderate or severe AI in HeartMate 3, as compared to HeartMate II, was 0.35 (*p* = 0 < 0.01, 95% CI 0.20–0.59) in their randomized study (46), which could be promising.

Thus, there are several conflicting studies to each other, regarding the risk factors of significant AI and its effect on mortality. However, given the results shown in the registry analysis (1), it would be reasonable to consider AV intervention at the time of CF-LVAD implantation in a patient with significant, or greater than mild, AI.

## Concomitant intervention on the aortic valve at CF-LVAD implantation

### Should we intervene on the AV with mild AI at CF-LVAD implantation?

Given these clinical impacts of late AI on the prognosis, some clinicians would advocate concomitant intervention on the AV at the time of CF-LVAD implantation, especially in patients of destination therapy. According to the recent ISHLT guideline, greater than mild AI (assessed by echocardiography with appropriate afterload) should be addressed with either valve closure, repair, or replacement (8) (Class of recommendations: I, Level of evidence: B). However, there is no definite consensus on whether we should preventively intervene on a competent AV with mild or less AI at CF-LVAD implantation. The recent reports on the efficacy of concomitant AV interventions at the time of continuous-flow LVAD implantation were summarized in Table 1. Based on the IMACS registry analysis, Veenis et al. reported that, even after adjustment for other significant predictors, concomitant AVR remained an independent predictor for early (HR 1.23, 95% CI 1.04–1.45) and late (HR 1.48, 95% CI 1.15–1.89) mortality (47). It should be noted, however, that when they focused just on the patients with moderate to severe AI, concomitant AV intervention was not an independent predictor of mortality, indicating that this result would not preclude concomitant AVR or repair when the patient has significant AI at CF-LVAD implantation. Likewise, based on the IMACS registry analysis, Yalcin et al. demonstrated that concomitant AV surgery at CF-LVAD implantation was associated with increased risk of bleeding events (HR 1.158, 95% CI 1.018–1.317, *p* = 0.026), but not thromboembolic events (48). These findings may indicate that stringent criteria for a concomitant AV procedure at the time of VAD surgery may be warranted, especially in patients with only mild AI (47).

**Table 1:** Efficacy of concomitant aortic valve interventions at the time of continuous-flow LVAD implantation.

**Study and design**	***N***	**Grade of AI**	**Results**	**Potential central messages**
Veenis et al. (47) and retrospective, IMACS	AVR (*n* = 457) AV repair (*n* = 328) No AV surgery (*n* = 14,482)	Overall Severe (0.7%) Moderate (3.8%) Mild (31.2%)	Concomitant AVR remained an independent predictor for early (HR 1.226, 95% CI 1.037–1.449) and late (HR 1.477, 95% CI 1.154–1.890) mortality. Patients undergoing AVR or repair for moderate/severe AI had survival similar to those without AV interventions.	Resolution of mild AI may not outweigh the risks associated with AV surgery, whereas resolution of moderate/severe AI may improve LVAD management.
Yalcin et al. (48) and retrospective, IMACS	AVR (*n* = 457) AV repair (*n* = 328) No AV surgery (*n* = 14,482)	Overall Severe (0.7%) Moderate (3.8%) Mild (31.2%)	Thromboembolic rate was 8% in AV surgery group and 9% in no AV surgery group. Concomitant AV surgery was an independent predictor for bleeding events.	Stringent criteria for a concomitant AV surgery at the time of CF-LVAD implantation may be warranted.
Fukuhara et al. (24) and retrospective, single-center	AV repair (*n* = 41) No AV surgery (*n* = 15)	Overall Mild (100%)	Freedom from AI >moderate at 2 years was 81.8% in AV repair group and 45.0% in No AV surgery group (*p* = 0.031). In No AV surgery group, 83.3% of patients with large body surface area-indexed aortic diameter developed >moderate AI, while none of the individuals with smaller aortic root did. In AV repair group, patients with large indexed aortic root have all been free of AI at 2 years.	While it is recommended that the AV be intervened on when the AI is more than mild, this study suggests that a subset of patients even with mild AI degree may benefit from an AV repair at the time of CF-LVAD insertion.
Fukuhara et al. (23) and retrospective, single-center	AV repair (*n* = 57) No AV surgery (*n* = 283)	Moderate/severe AI by group AV repair (24.6%) No AV surgery (0%)	Kaplan-Meier analysis revealed that Freedom from significant AI was 66.7% in AV repair group and 59.9% in No AV surgery group at 2 years (*p* = 0.77). A generalized mixed-effects model demonstrated a 57% decrease in the odds of significant AI progression in AV repair group, after adjusting for time effect and degree of baseline AI.	Concomitant AV repair may be an effective strategy in addressing pre-existing AI for patients support by CF-LVAD.
Robertson et al. (51) and retrospective, INTERMACS	AVR (*n* = 85) AV repair (*n* = 95) AV closure (*n* = 125) No AV surgery (*n* = 5,039)	Moderate/severe AI by group AVR (47.8%) AV repair (38.8%) AV closure (35.7%) No AV surgery (2.0%)	After adjustment, AV closure was an independent predictor of mortality (HR 1.87, 95%CI 1.39–2.53, *p* < 0.0001). At 6–12 months post-operatively, moderate to severe AI developed in 19, 5, 9, and 10% of patients who underwent AV repair, AV closure, and AVR and No AV surgery (*p* < 0.0001).	Concomitant AV repairs maybe performed during CF-LVAD implantation with results comparable to those for patients who did not undergo AV repair. AV closure is associated with significant reductions in both short- and long-term mortality. The durability of an AV repair, however, is worse than for other approaches.
McKellar et al. (22) and retrospective, single-center	AV repair (*n* = 18) No AV surgery (*n* = 105)	Greater than mild AI by group AV repair (100%) No AV surgery (0%) AI score (0–5) AV repair (1.8 ± 1.4) No AV surgery (0.15 ± 0.43)	At median follow-up of 312 days, the mean AI score remained lower for AV repair group (0.27 ± 0.46) than that for No AV surgery group (0.78 ± 0.89, *p* = 0.02). The proportion of patients with more than mild AI was significantly less in AV repair group (0 vs. 18%, *p* = 0.05) The patients in AV repair group were significantly older and had a greater incidence of renal failure at baseline.	AV repair using a central coaptation stitch is effective in reducing AI in patients with native valve AI at CF-LVAD implantation. Longer term follow-up is required to determine whether its use is warranted prophylactically in patients of destination therapy.
			The 30-day mortality was greater in AV repair group, but the late survival was similar between the two groups. No reoperations were required for recurrent AI.	
Tang et al. (28) and retrospective, single-center	AV repair (*n* = 40) AVR (*n* = 6)	Moderate/Severe AI by group AV repair (70.0%) AVR (66.7%)	In AV repair group, AI severity was decreased by 2.1 ± 1.0 grades (*p* < 0.001), but 7.5% had recurrence of at least moderate AI by 3 years. Success of AV repair in downgrading AI severity was associated with a smaller aortic root diameter (*p* = 0.011) and sinotubular junction diameter (*p* = 0.003). Duration of cardiopulmonary bypass was 32 min longer and duration of aortic cross-clamp time was 38 min longer for AVR vs. AV repair group. No difference in 30-day or overall survival between AV repair and AVR group was seen.	AV repair at CF-LVAD implantation is efficacious and durable. AI recurrence rate of 7.5% at 3 years represents a reasonable compromise between its simplicity and expediency vs. durability. Alternatively, a bioprosthetic AVR can be performed.
Kurihara et al. (29) and retrospective, single-center	LVOT closure (*n* = 16) No LVOT closure (*n* = 510)	Severe AI by group LVOT closure (68.8%) No LVOT closure (0%)	Survival at 30 days, 6 months, 1 year, and 2 years was similar for No LVOT closure group (90.4, 80.6, 74.3, and 67.5%) and LVOT closure group (81.3, 81.3, 75.0, and 68.8%, *p* = 0.59). There were no deaths related to LVOT closure.	For select patients with AI who are undergoing CF-LVAD implantation, LVOT closure produces acceptable outcomes and, therefore, is a viable option. Longer-term studies are necessary to determine whether aortic root thrombus and subsequent thromboembolic complications eventually become an issue in these patients.

N, Number of patients; AI, Aortic insufficiency; AV, Aortic valve; AVR, Aortic valve replacement; HR, Hazard ratio; CI, Confidence interval.

On the contrary, while there are several concerns for concomitant AV procedures, Tanaka et al. reported the detrimental impact of uncorrected mild AI at the time of CF-LVAD implantation on the non-survival outcomes (49). Although their analysis was a single-center one and did not demonstrate significant survival differences, after propensity-score matching, uncorrected mild AI was significantly associated with a higher risk of progression to moderate or greater AI (43.6% with the mean follow-up period of 2.3 ± 1.8 years) and worse NYHA functional class (*p* < 0.01). Notably, more CHF-related readmissions were observed in the mild AI group, as compared with no or trace AI (HR: 2.62, 95% CI 1.42–4.69) (49). Their results shed light on the need for proactive intervention on the mild AI at CF-LVAD implantation to improve the patients' quality of life in the future.

As for the surgical management of mild AI, Fukuhara et al. reported the efficacy of concomitant AV repair, or central AV closure as described later, at CF-LVAD implantation on the progression of AI (24). This study by Fukuhara is unique and worthwhile, in that they specifically focused on those with mild AI, to reveal whether we should intervene on the AV with mild AI simultaneously at VAD implant or not. In the AV repair group, freedom from AI greater than moderate at 2 years was 81.8% as compared to 45.0% in the AV non-repair group (*p* = 0.031), leading to no survival difference (24). Interestingly, their decision to perform a repair was made on the selected candidates with anticipated prolonged device support, such as destination therapy, bridge-to-transplant patients with large body size (body mass index >35) and bridge-to-transplant patients with blood type O (50). Given the recent refinements in surgical technique and expected longer waiting period in heart transplant candidates, the threshold of intervening on the AV would be gradually getting lower, especially in these selected candidates.

### What is a desirable concomitant AV intervention at CF-LVAD implantation, AVR or AV repair?

An ideal AV procedure to treat AI, simultaneously with CF-LVAD implantation, is still controversial. Potential options could be AVR, AV repair, and AV closure. Based on the INTERMACS registry analysis, Robertson et al. reported that actuarial 1-year survival after CF-LVAD implantation was significantly worse in those who underwent concomitant AV closure (AV closure vs. AV repair vs. AVR, 63.2 vs. 76.8 vs. 71.8%, *p* = 0.0003) (51). As for the efficacy of AI treatment, they also demonstrated that AI recurrence rate (moderate to severe) at 6 to 12 months after the implantation was the highest in the AV repair group (AV closure vs. AV repair vs. AVR vs. No intervention, 5 vs. 19 vs. 9 vs. 10%, *p* < 0.0001) (51). Although Kurihara et al. reported the feasibility of AV (or left ventricular outlet) closure as a concomitant first-line procedure at CF-LVAD implantation, a disadvantage to close AV is that the patient will not be able to maintain hemodynamic stability if the device fails, and that bridge to recovery is no longer an option, as they admit (29). From these results, it would be reasonably safe to avoid AV closure from the first-line modalities.

While AVR with bioprosthetic valve contains a few issues, such as longer ischemic time, valve thrombosis, or commissural fusion (52), AV repair would be advantageous in terms of these issues, at the expense of potential recurrent AI in the future. As there are several other different techniques of AV repair in VAD-associated AI, for example aortic ring annuloplasty (25, 26), one of the most typical techniques among AV repair is a central AV closure (CAVC), or what we call Park's stitch, which was originally reported by Park et al. for a pulsatile LVAD in 2004 (21). This is basically a technique for central AI without any structural problems on the AV, by putting a simple coaptation stitch with a pledged supported 4-0 polypropylene sutures to approximate the fibrous nodules of Arantius. As compared to AV closure, the benefit of CAVC is that AV is still able to open for ejection, even though the effective orifice area is diminished. CAVC has the potential to be the ideal technique because it is inexpensive, quick, and simple to perform, and might not have the same degenerative potential as biologic valve prostheses. In 2014, Park's group first published its efficacy in CF-LVADs (22). They conducted a concomitant CAVC at the time of CF-LVAD implantation in 18 patients, those with greater than mild AI at baseline. Amazingly, among all the 18 patients, the grades of AI were mild or less at 2 years after CF-LVAD implantation (22).

A largest single-center experience of AV repair by central AV closure at VAD implantation is reported by Fukuhara and colleagues (23). They conducted concomitant central AV closures in 57 patients at the time of CF-LVAD implantation and its efficacy was compared with 283 patients those who underwent CF-LVAD implantation without central AV closures. Although Fukuhara et al. did not find any significant survival differences between the groups, their generalized mixed-effects model demonstrated a 57% decrease in the odds of significant AI progression among those who underwent the central AV closure as a concomitant procedure, after adjusting for time effect and degree of baseline pre-existing AI (23).

Thus, while CAVC could potentially be a first-line treatment of AI at the time of CF-LVAD implantation, one of its major drawbacks is a recurrence of AI during follow-up. Although there are few studies directly comparing CAVC with AVR, Tang et al. conducted a retrospective analysis on the concomitant CAVCs (*n* = 40) and AVRs (*n* = 6) (28). The CAVC group yielded shorter ischemic and cardiopulmonary bypass time, however, 7.5% of CAVC patients had recurrence of at least moderate AI by 3 years. Although they did not find any survival difference between the groups, such a decision of CAVC or AVR, as a concomitant procedure at CF-LVAD implantation, should depend on each clinical context.

## The effect of impella—Another indispensable consideration

In 2018, the new United Network for Organ Sharing (UNOS) donor heart allocation system commenced giving a priority to patients supported with non-dischargeable mechanical circulatory support (MCS) devices while awaiting heart transplantation, prompting temporary MCS devices in heart transplant centers being more frequently used in the United States (US) (53). The Impella device (Abiomed Inc, Danvers, MA, USA) is an axial-flow percutaneous ventricular assist device used in cases in cardiogenic shock (54), and the use of Impella is gradually increasing especially in the US, reflecting the forementioned updates in the UNOS criteria. As for those who are eligible for heart transplantation, recently they could be directly bridged to transplant with the Impella in the US, as a new status 2 category. However, those who are ineligible for transplant might need to undergo CF-LVAD implantation as destination therapy to survive, and there are several reports on the adverse impact of the Impella on the AV in such patients (55–58). In fact, just on the Impella support, an increase in AI grade was observed in 17.2% of patients with an event per support days of 0.03 (55). Such a potentially iatrogenic damage on the AV could be associated with AI development even after VAD implants. Rao et al. compared the development of *de novo* AI after CF-LVAD implantation between those who were on the Impella support and those not, concluding that mild or moderate *de novo* AI was observed in 82% of patients in the Impella group, as compared 43% in the non-Impella group (*p* = 0.038) (56). The pathophysiology of AI due to the Impella support is still unclear. Oishi et al. reported two cases of *de novo* moderate AI due to the Impella, both of which required concomitant CAVCs at the VAD implantation (57). They speculated, like CF-LVAD support, that the AV is not opening by the Impella support, making the pressure load on the AV greater and causing disorganization and remodeling of the valve (57). Thus, especially when the Impella was placed before CF-LVAD implantation, careful intraoperative observation of the AV is mandatory at VAD implant, of course after the Impella removal, to avoid future progression of Impella-induced *de novo* AI.

## Preventive strategies of *de novo* AI after CF-LVAD implantation

Up to this point, we summarized the current updates of AI associated with CF-LVAD, regarding its pathophysiology, incidence, clinical significance, and its concomitant surgical treatment at the time of CF-LVAD implantation. Henceforth, we moved on to the preventive strategies of *de novo* AI after VAD implants (14), followed by the options of late intervention on the AV.

Since continuous closure of the AV is reported as one of the major risk factors of *de novo* AI (17, 37, 59, 60), pump speed optimization to maintain AV opening would be one of the key aspects in the prevention of *de novo* AI (16). Jorde et al. demonstrated the efficacy of speed optimization study, or right heart catheter pressure study with transthoracic echocardiography with different pump speed before discharge, on the prevention of *de novo* AI (59). They conducted this optimization in 29 patients, and they found only 1 patient developed greater than mild AI during a median follow-up time of 205 days (59). In contrast, without this optimization study, 20 out of 62 patients developed greater than mild AI during a median follow-up time of 265 days. Jorde et al. concluded that their speed optimization study before discharge was significantly associated with reduced risk of *de novo* AI after CF-LVAD implantation (HR 11.2, 95% CI 4.6–27.4, *p* = 0.003) (59). Based on their report, such a speed optimization study is now routinely performed before discharge in some VAD-implant centers.

Another key aspect in the prevention of *de novo* AI could be afterload optimization. In fact, in non-VAD general populations, elevated systolic pressure is known to be associated with increased risk of valvular heart disease including AI (61, 62). Mechanistic evidence for the potential causal role of high blood pressure on *de novo* AI is unclear, although some speculate that high blood pressure causes abnormally high tensile stress on the AV, which can lead to endothelial injury or disruption (62). Like general populations, blood pressure management is an essential part of the routine care of CF-LVAD patients, especially for the prevention of thromboembolic events and *de novo* AI. Patil et al. reported that systolic blood pressure at 3 months after CF-LVAD implantation was an independent predictor of more than mild *de novo* AI, as well as aortic valve closure and longer support durations (60). However, in other studies, such a significant association between blood pressure in CF-LVAD patients and AI development was not observed (17, 59). Table 2 shows the summary of suggested medical and surgical interventions to treat AI in the patients support by CF-LVAD. Thus, so far there is no established strategies that can perfectly prevent the progression of AI. Once *de novo* AI in CF-LVAD patients becomes significant, next we need to consider when to intervene.

**Table 2:** Summary of suggested medical and surgical interventions to treat aortic insufficiency in patients support by continuous-flow LVAD.

**Timing**	**Interventions**	**Benefits**	**Risks**
Timing 1: At CF-LVAD implantation Better to intervene if greater than mild AI is seen For mild AI, it depends on the expected support duration or AV morphology.	AV repair	Shorter ischemic time	Potential recurrence of AI
	Bioprosthetic AVR	Longer durability	Longer ischemic time
	AV closure	Shorter ischemic time Longer durability (potentially)	Potentially thrombogenic Difficulty in LVAD weaning
Timing 2: During CF-LVAD support Medical managements to avoid worsening of AI Significant AI often requires high pump speed for compensation.	Speed optimization (right heart catheter or echo guided)	Avoid continuous closure of AV	Inappropriate speed may cause under-supported condition
	Afterload adjustment (vasodilator)	Reverse flow to left ventricle may decrease	Hypotension
	Volume optimization (intake restriction or diuretics)	Reverse flow to left ventricle may decrease	Low output syndrome
Timing 3: When significant AI refractory to medical managements is seen	AV repair	Shorter ischemic time	Potential recurrence of AI
	Bioprosthetic AVR	Longer durability	Longer ischemic time
	TAVI	Less invasive	Valve migration Paravalvular residual AI
	Transcatheter AV closure	Less invasive	Potentially thrombogenic Difficulty in LVAD weaning

CF, continuous-flow; AV, Aortic valve; AVR, Aortic valve replacement; TAVI, Transcatheter aortic valve implantation.

## Late intervention on the aortic valve

Even today, there is no definite consensus on when to intervene significant *de novo* AI after CF-LVAD implantation (16). Clinically, severe AI does not necessarily result in heart failure or elevated filling pressures. First-line medical treatment of *de novo* AI could be diuretics and vasodilators to decrease congestion and control blood pressure. However, once the patient becomes symptomatic because of significant AI, he or she surely needs to undergo right heart catheter study with simultaneous echocardiography for speed optimization (63). An increase in pump speed might be considered to improve cardiac output and end-organ perfusion, but this is at the expense of worsening AI. In general, this speed optimization for significant AI is only palliative and effective in the short term. Even today, there is no clear recommendation regarding the most pertinent surgical or interventional options to treat such patients. If the patient is eligible for heart transplantation, upgrading on the waiting list could be considered in some countries. Other potential treatment modalities are like the ones mentioned previously at the concomitant procedures with CF-LVAD implantation (15). Those are bioprosthetic AVR, CAVC, and surgical AV closure. In the future, total artificial heart could be another choice (64). Since these options requires redo-sternotomy in such a high-risk patient with elevated filling pressures due to significant AI, TAVI or trans-catheter device closure of AV can also be a reasonable select for *de novo* AI.

## What is a desirable secondary AV intervention for late *de novo* AI during CF-LVAD support, AVR, AV repair, or else?

To the best of our knowledge, prospective studies on the efficacy of bioprosthetic AVR or AV repair for late *de novo* AI is quite limited, probably because of the following three reasons: (1) we currently tend to intervene on the AV more aggressively at the time of CF-LVAD implantation and the need of late AV intervention is decreasing, (2) for late *de novo* AI, less invasive procedures, such as TAVI or percutaneous closure, are more likely to be conducted instead of surgical interventions, and (3) In some countries, urgent heart transplantation is now becoming a feasible option to deal with *de novo* significant AI. For these reasons, there is no definite agreement on an ideal secondary AV intervention, and the decision should depend on each clinical scenario.

Nonetheless, AVR with bioprosthetic valve could be a gold standard therapy of *de novo* AI in VAD patients, especially when significant morphological change in the AV is observed. However, this procedure necessitates longer ischemic time and decent exposure of the aortic root, as compared to AV repair, which may raise a concern for postoperative right ventricular dysfunction in these high-risk candidates (19). We found two case series reports on the secondary AVR for late *de novo* AI (19, 20). Firstly, Atkins et al. reported that 6 out of 225 CF-LVAD patients developed *de novo* severe AI accompanied by heart failure, and for these 6 patients, they conducted 1 AVR with bioprosthetic valve, 1 Dacron patch closure, 2 aortic valve repair, and 2 TAVIs, one of which required revision by open surgery for AVR (20). Among these 6 patients, while 5 patients experienced significant improvement in functional capacity and symptom, 1 patient who underwent AVR unfortunately passed away postoperatively secondary to multiorgan failure and sepsis (20). Secondly, Gyoten et al. reported the similar case series of late AVR for *de novo* AI (19). They performed a total of 792 CF-LVAD implantations during the study period, and among them, 6 AVRs were performed for late severe AI, all of which were successfully done. However, 4 patients required temporary right ventricular assist devices, and 3 of them necessitated urgent heart transplantation to survive right heart failure. Judging from these two reports by Atkins and Gyoten (19, 20), secondary AVR for *de novo* AI in CF-LVAD patients is surely a procedure with considerable surgical risks.

A next question here would be whether AV repair can be a satisfactory alternative of AVR, with less risks and comparable outcomes. As far as our investigation, unfortunately, there is few reports on the efficacy of AV repair for late *de novo* AI. Our group previously published one case report of AV repair, or CAVC, for late *de novo* AI in a CF-LVAD patient, which was quite successful (27). Certainly, AV repair is less invasive as compared to AVR, in that it merely needs shorter ischemic time and less dissection around the aortic root, at the expense of potential AI recurrence in the future. Although we do not find the evidence on long-term outcomes after secondary AV repair for late *de novo* AI, based on the AI recurrence rate after concomitant AV repair at CF-LVAD implantation (23, 24, 51), clinical utility of AV repair could be similar to AVR.

Apart from the clinical case series above, there is a registry database analysis on the risk of AVR in CF-LVAD patients. Zaidi et al. reported the survival outcomes of AVR and TAVI late after CF-LVAD implantations, using the Nationwide Readmission Database in the US (7). Although they did not refer to the efficacy of AVR or TAVI in terms of controlling AI grade, they demonstrated in-hospital mortality was significantly higher in the AVR group than the TAVI group (42.3 vs. 6.4%, adjusted OR 10.4, 95% CI 1.37–79.5, *p* = 0.02), warranting a prudent judgement on the indication of surgical AVR for late *de novo* AI. Additionally, Doi et al. reported a case of commissural fusion after bioprosthetic AVR after CF-LVAD implantation, casting doubt on bioprosthetic AVR as a desirable option, as compared to AV repair (52). Right ventricular failure due to longer ischemic time is another non-negligible concern, as Gyoten et al. reported (19). Based on these data, the decision of AVR, AV repair, or other AV interventions should be tailored by case-by-case basis, considering surgical risks, right ventricular functions, AV morphologies, and expected support time.

## TAVI is less invasive, but still not a promising option

Another option for treating AI could be TAVI. Although TAVI is not used routinely as a treatment option for severe AI, as of the year 2022, an international multicenter registry data already demonstrated its feasibility and efficacy in non-VAD patients, especially with new-generation devices (65). However, when it comes to the AI on CF-LVAD patients, only a few case series with very limited sample size (30, 32, 33, 66) or single case reports (67–72) are found. Yehya et al. conducted a TAVI in 9 CF-LVAD patients for severe AI (30). They reported all the 9 patients were discharged home and 8 patients were alive at 6 months. Five procedural complications were found, which are two valve migrations, one retroperitoneal hematoma, one groin hematoma, and one femoral pseudoaneurysm (30). As for two cases complicated with valve migrations, they used CoreValve 31 mm (Medtronic, MN, USA), one of the prior generation devices. Technically, most of TAVI devices are not initially designed to place on the dilated annulus in such AI patients. As Yehya et al. admits, in AI cases the lack of significant annular calcification to serve as an anchor for the valve can pose a technical challenge while increasing the risk of valve migration and lack of stability (30). In fact, even in the centers of excellence with new-generation devices, the second valve implantations were required in 12.7% of pure AI patients who underwent TAVI (65). Additionally, suction from the *in-situ* CF-LVAD may disturb prosthesis deployment and increase the risk of prosthesis migration and still there is no consensus on how to optimize pump speed to prevent valve migration while deploying the device (69). Hopefully, these issues may be partly addressed by the devices technically designed to place on the annulus without significant calcification (31, 73), such as JenaValve (JenaValve Technology, Munich, Germany) (70)and J-valve (JC Medical, Suzhou, China).

There are three other case series regarding TAVI for AI in CF-LVAD patients. First one is by Belkin et al., reporting 7 patients underwent 9 attempted TAVI procedures (33). Unfortunately, two patients expired within the first day for cardiogenic shock due to inadequate valve fixation and severe paravalvular leakage. Five patients out of 7 (71%) survived over median follow-up of 9 months. It is noteworthy that they demonstrated significant improvements in the right ventricular function, as well as the degree of AI (33). Second one is by Gondi et al., reporting 11 patients underwent TAVI. Like the report by Belkin et al., one died during the procedure from ventricular fibrillation associated with valve migration and one died 19 days after the procedure for persistent shock. Eight patients out of 11 (73%) were alive at 12 months, and all survivors had improvement in the grade of AI and NYHA class (66). Third one is by Dhillon et al., reporting 4 patients underwent TAVI (32). One valve migration occurred out of 4 cases, which required a rescue valve-in-valve procedure. Although all the 4 patients were once successfully discharged home, 3 patients (75%) expired at 10 days, 2 months, and 3 months after the procedure, by congestive heart failure, septic shock, and LVAD thrombosis, respectively (32). These data might indicate that their TAVI candidates could have been ineligible for redo surgical intervention, just because too sick at baseline, and accordingly their outcomes after TAVI, possibly a palliative option, was still quite poor. Thus, TAVI could be a reasonable option for the treatment of AI in selected CF-LVAD cases, however, prospective studies with larger sample size are needed to assess the durability and long-term efficacy of this procedure, in addition to its technical refinements.

## Trans-catheter closure of the aortic valve; is a last option to consider?

As mentioned previously, concomitant AV closure at CF-LVAD implantation was associated with increased risk of mortality (51). Moreover, it also contains ineluctable drawbacks, such as risk of sudden death if the device fails, and difficulty in CF-LVAD weaning even when recovery is an option (29). In this sense, AV closure, surgical or trans-catheter one, could be a last option to consider. However, same as TAVI, for those who cannot tolerate invasive open surgery, such as old CF-LVAD patients of destination therapy, trans-catheter closure of the AV might be a palliative option to treat AI under selected circumstances. Retzer et al. reported the efficacy of trans-catheter AV closure using an Amplatzer Multi-Fenestrated Septal Occluder “Cribriform” device to close the AV of CF-LVAD patients (34). Notably, technical success was accomplished in 100% of patients. However, 6-month survival rate was only 30%, reflecting pre-procedural co-morbidities such as right ventricular failure. Phan et al. conducted a systematic review and meta-analysis on the outcomes after percutaneous trans-catheter interventions for AI in CF-LVAD patients (35). They included 8 cases of TAVI and 21 cases of trans-catheter AV closure, concluding that both procedures were effective in reducing the AI grade. Nonetheless, while 20 months survival was ~35% in the TAVI group, it was zero in the trans-catheter AV closure group. These data might indicate that survival outcomes after trans-catheter AV closure are unsatisfactory. Therefore, this option might not be the first-line treatment of VAD-associated AI, especially in young and healthy candidates. Furthermore, the ethical dilemma of AV closure with considerations of CF-LVAD withdrawal is another important consideration. Even on the appropriate level of sedation, sudden termination of pump rotation may lead to immediate death associated with acute pulmonary congestion in the patients with AV closure. In view of comfort care at terminal stage, we cannot overlook such an ethical drawback of AV closure.

## Conclusions

The present review summarized current updates on CF-LVAD associated AI, in terms of its pathophysiology, incidence, clinical impacts on outcomes, prevention, and surgical or trans-catheter interventions. As a concomitant procedure with CF-LVAD implantation, current guidelines are recommending AV repair or AVR for greater than mild AI, which is well-supported (15). For mild or less AI at VAD implants, the decision to intervene on the AV should be tailored by case-by-case basis, considering patients' co-morbidities, surgical risks, right ventricular functions, AV morphologies, and expected support time. Correcting mild AI during CF-LVAD implantation may be reasonable in destination therapy patients. As for the managements of late *de novo* AI, still there is no clear consensus on the timing of intervention or the choice of treatment modality. Clearly, symptomatic severe AI in a CF-LVAD patient needs to be addressed, either surgically or percutaneously. Hopefully in the future, TAVI would become a first-line treatment of late *de novo* AI in CF-LVAD patients, after technical refinements and device improvements. Despite the scarcity of established evidence so far, our continuing efforts are imperative to develop new insights in the future, overcoming this scabrous clinical entity during CF-LVAD support.

## Author contributions

Both authors listed have made a substantial, direct, and intellectual contribution to the work and approved it for publication.

## Conflict of interest

The authors declare that the research was conducted in the absence of any commercial or financial relationships that could be construed as a potential conflict of interest.

## Publisher's note

All claims expressed in this article are solely those of the authors and do not necessarily represent those of their affiliated organizations, or those of the publisher, the editors and the reviewers. Any product that may be evaluated in this article, or claim that may be made by its manufacturer, is not guaranteed or endorsed by the publisher.

## Abbreviations

ISHLT: international society for heart and lung transplantation
INTERMACS: interagency registry for mechanically assisted circulatory support
AV: aortic valve
AVR: aortic valve replacement
HR: hazard ratio
OR: odds ratio
CI: confidence interval
NYHA: New York Heart Association
CHF: congestive heart failure
CAVC: central aortic valve closure.
